# Effectiveness of intratracheal salbutamol in addition to surfactant on the clinical course of newborns with respiratory distress syndrome: a clinical trial

**DOI:** 10.1186/s13052-016-0215-1

**Published:** 2016-01-19

**Authors:** Masoud Dehdashtian, Arash Malakian, Mohammad Reza Aramesh, Ali Mazori, Mohammad Hasan Aletayeb, Afsaneh Shirani, Shiva Bashirnejad

**Affiliations:** Pediatrics Department, Imam Khomeini Hospital, Ahvaz Jundishapur University of Medical Sciences, Ahvaz, Iran

**Keywords:** β agonist, Premature infants, Continuous positive, Airway pressure, Respiratory distress syndrome, Surfactant

## Abstract

**Background:**

In addition to surfactant deficiency, increase of lung fluid content and secretion of fluid derived from the blood participate in the pathogenesis of RDS in newborns.

We hypothesized that the administration of salbutamol (β-agonist) to increase lung fluid absorption would decrease the INSURE failure rate in newborns with respiratory distress syndrome (RDS) treated with intratracheal surfactant.

**Methods:**

**Design**

Blinded, randomized clinical trial study.

**Setting/population**

Level III NICU, premature infants with RDS requiring intratracheal Surfactant.

Forty Eight newborns with RDS treated with intratracheal Surfactant were randomized into two groups as Group A, Normal saline (as control group) and Group B (intervention group), Salbutamol were administered intratracheally in addition to Surfactant. Intubation-Surfactant administration- Rapid Extubation (INSURE) failure rate as primary outcome and secondary outcome as follow: duration of the need to NCPAP, mechanical ventilation and oxygen therapy; complications (patent ductus arteriosus, pneumothorax); mortality (respiratory or prematurity related complication) and the duration of hospitalization were assessed.

**Results:**

Twenty Four patients in each group were studied. INSURE failure was seen in16 (66.7 %) and 10 (41.7 %) of normal saline and salbutamol groups respectively (*p* = 0.082). The duration of NCPAP in control group was 69.5 ± 54.9 h while in Salbutamol group was 51.6 ± 48.7 h (*p* = 0.316). All of deaths were related to respiratory failure. No differences in mortality or complications of RDS were observed. The duration of hospitalization was longer in control group than interventional group, 28.3 ± 18.1 and 18.6 ± 8.6 days, respectively. (*p* = 0.047).

**Conclusion:**

Salbutamol may improve the clinical course of newborns with RDS requiring Surfactant.

**Trial registration number:**

IRCT2014072714215N1

## Background

Respiratory distress syndrome (RDS) is one of the most common causes of neonatal respiratory failure and neonatal mortality. The risk of developing RDS increases with decreasing gestational age. Immaturity of the lungs and, specifically, surfactant deficiency are the cornerstone of RDS in newborns [1]. In addition to Surfactant deficiency, increase of lung fluid content and secretion of fluid derived from the blood participate in the pathogenesis of RDS in newborns [2]. During the last gestation period, the fetal lung airways contain 40 ml/kg fluid which is somewhat greater than the Functional residual capacity [3]. Active transport of chloride ion from the plasma into the pulmonary alveolar spaces creates an electrical potential difference of 4.3 mV between lung liquid and plasma [4]. The osmotic force that is generated by this secretion process results in the transport of fluid from pulmonary microcirculation and interstitial fluid into alveolar spaces. Active transport of clˉ from plasma into alveolar spaces is probably mediated via Na-K-2Cl (NKCC) co-transporters [5].

Transport of clˉ that begins in mid-gestation is inhibited by loop diuretics such as Furosemide that block NKCC co-transporters [6].

Labor process has an important role in lung airways fluid absorption and preparation for air respiration. During vaginal delivery, a lot of lung fluid propelled from the airways and mouth, but the active transport of sodium mediated via Na^+^/K^+^-ATPase channel is the basic mechanism for the clearance of fetal lung airways [7–10]. Several investigators have shown that high endogenous catecholamine secretion at birth is an important factor in fetal lung clearance by the induction of Na^+^/K^+^-ATPase pump [11, 12]. In addition, it has been shown that β-agonists increase the Na channel activity mediated by cAMP-PKA and lung sodium transport [13]. Secretion of liquid into the trachea of the fetal lambs begins to decrease a few days before spontaneous vaginal delivery [14]. Very premature infants have immature lungs that are surfactant deficient, partially liquid filled, and prone to atelectasis at end expiration [15].

Mechanical ventilation (MV) and nasal continuous positive airway pressure (NCPAP) are the most respiratory support techniques that used in treatment of newborns with RDS [16]. Intubation-Surfactant administration- Rapid Extubation (INSURE) is associated with less need to mechanical ventilation, lower incidence of BPD and fewer air leak syndromes [17]. Although this method has been clearly associated with reduced need for MV [18] not all infants can be successfully managed with this modality. INSURE failure has been registered in 27 to 35.6 % [19, 20].

Our objective in this study was to assess the effects of Salbutamol in order to increase fluid absorption from the alveoli and reduction of INSURE failure in premature newborns with RDS.

## Methods

This clinical trial study with registration number IRCT2014072714215N1 was performed at Neonatal intensive care Unit of Imam Khomeini Hospital, Ahvaz Jundishapur University of Medical Sciences, Ahvaz, IRAN, a teaching tertiary care center with 36 and 45 beds of level 3 and 2 respectively. Initially as a pilot study, 10 newborns with respiratory distress syndrome who needs intratracheal surfactant beside NCPAP management were randomly assigned to receive surfactant plus normal saline or surfactant plus salbutamol. The sample size was calculated based on our primary outcome (insure failure), p1: 0.4, p2: 0.08, considering 80 % as the power of the study, and confidence interval 95 %. As the results, 24 patients in each group were enrolled in this study.

Newborns at our hospital with gestational age less than 34-o/7 weeks and birth weight greater than 1000 g., with a diagnosis of RDS according to clinical and radiographic findings and negative blood culture were eligible for inclusion in this study. Because of high mortality rate of newborns with birth weight less than 1000 g. in our institute, they did not included in this study. The clinical signs and symptoms of RDS were respiratory distress, tachypnea, nasal flaring and grunting after birth. The typical chest x- ray picture of RDS showed a grainy shadow, air bronchogram, and white lung. Newborns with birth trauma, congenital anomalies, and perinatal asphyxia, 5 min apgar score ≤ 3, negative radiologic finding, severe metabolic acidosis at birth and positive blood culture were excluded. Patients were hospitalized at our neonatal intensive care unit from March 21, 2014, through July 11, 2014. An informed consent was obtained from patients’ parents. Newborns diagnosed with RDS and birth body weight greater or lesser than 1500 g were treated with oxyhood or nasal continuous positive airway pressure (NCPAP) [21]. Arterial Blood Gases (ABG) was measured 30 min later. Neonate with ABG (Pao2: 50–70 mmHg, pH: 7.25- 7.45, Paco2: 40–50 mmHg) were observed closely. Under NCPAP neonates with ABG (pH 7.3- 7.45, Paco2 40–50 mmHg, Pao2 < 50 mmHg) while received 6 cm H2o PEEP and needed greater than 40 % Fio2 were treated with intratracheal surfactant [20]. NCPAP were discontinued in infants with favorable ABG when received 4 cm H2o PEEP and Fio2 lesser than 40 % without increasing work of breathing, no substantial apnea and bradycardia [22].

Surfactant was administered to the infants base on INSURE protocol within 2 h of birth. Curosurf (Cheisi farmaceutici S.P.A) with initial dose of 2.5 ml/kg and subsequent doses of 1.5 ml/kg were used [23]. Patients who required to Fio2 greater than 0.3 were treated with repeated doses of surfactant.

The neonates treated with Surfactant and Normal saline (control group) or Salbutamol (interventional group) base on the computerized randomization list. In addition to Curosurf, the doses were used as follows: intratracheal Normal saline of 0.5 ml/kg or intratracheal Salbutamol of 0.2 mg/kg [24]. Solution containing Salbutamol 0.4 mg/ml was prepared with clinical pharmacist. Normal saline was used for the dilution of Salbutamol and preparation of suitable volumes. Therefore, in each patient group, a volume equal to 0.5 ml/kg of Normal saline or prepared solution was administered in addition to Surfactant. Normal saline or Salbutamol was administered immediately after administration of Surfactant via the same intratracheal tube. In each patient, if additional doses of Surfactant were needed, the same solution was used again. Solution vials containing Normal saline and Salbutamol were labelled with A and B, respectively. Echcardigraphy was performed in newborns patient with hyperkinetic heart, murmur, binding pulses and increased need to respiratory support after third days of life. The physician and caretaker were blinded to vial content, and the patients were enrolled in the study according to the table of computerized randomization list.

## Primary and secondary outcomes

The primary outcome was INSURE failure. INSURE failure was defined as the need for reintubation and MV within 72 h of extubation. Mechanical ventilation was considered in babies with PaO_2_ < 50 mmHg or PaCO_2_ > 55 mmHg and pH < 7.25 with FiO_2_ > 0.4 and PEEP > 6 cm H2o; or those with increasing work of breathing including severe intercostal retractions on PEEP >7 cm H2o; or prolonged (>20 s) or recurrent apneas and bradycardia (>2 episodes within 24 h) need to bag and mask ventilation [25, 26]. Ventilated newborns with suitable ABG (Pao2 60–80 mmHg, Paco2 40–55 mmHg and pH 7.25- 7.45) and without increasing work of breathing were moved to NCPAP when they received a low PIP (10–12 cm H2o), Fio2 lesser than 40 %, and respiratory rate of 10- 15/min [27]. The secondary outcomes; duration of the need to NCPAP, Mechanical Ventilation, oxygen and also mortality (respiratory or prematurity related complication), morbidity (patent ductus arteriosus, pneumothorax) and discharge from hospital were assessed. Full oral feeding (150 ml/kg/day) and starting to weight gain was the discharge criteria.

## Statistical analysis

Comparison between continuous and independent variables was performed using Mann–Whitney, and chi-square test. All the statistical analysis was performed using SPSS version 16 (IBM, Armonk, New york). *p* Value <0.05 was considered significant.

## Results

**Fig. 1:**
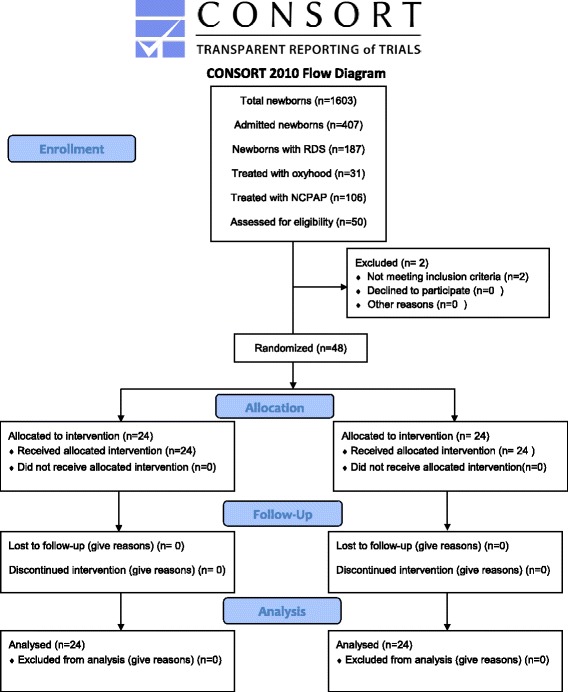
Participant CONSORT flow diagram: approach to neonates with respiratory distress syndrome treated with intratracheal surfactant and salbutamol or only surfactant

Of the 50 patients enrolled in this study, one patient in control group and another in Salbutamol group were excluded from the study when diagnosed with perinatal asphyxia and congenital heart disease (Fig. 1).

Demographic data of studied patients have been shown in Table 1. Tachycardia (heart rate > 160/min) was not seen in any neonate treated with salbutamol.

**Table 1:** Patients’ demographic characteristics

Variable	Surfactant with N.S. (*n* = 24)	Surfactant with Salb. (*n* = 24)	*p*
G. age (week)	29.70 ± 2.27	30.04 ± 2.07	0.59
B.B.W (g)	1430.83 ± 393.41	1443 ± 267.22	0.89
Gender			
M.	15(62.5 %)	17(70.8 %)	0.54
F.	9(37.5 %)	7(29.2 %)	
Delivery			
C.S.	18(75 %)	19(79.2 %)	0.73
Vag.	6(25 %)	5(20.8 %)	
Perenatal steroid			
Yes	11(45.8 %)	10(41.7 %)	0.77
No	13(54.2 %)	14(58.3 %)	
Curosurf dosage			
1	20(83.3 %)	18(75 %)	0.47
2	4(16.7 %)	6(25 %)	

*N.S* normal saline, *Salb* salbutamol, *G* gestational, *B.B.W* birth body weight, *M* male, *F* female, *C.S* cesarean section, *Vag* vaginal

NCPAP failure rate and the need for mechanical ventilation were greater in control group (Table 2). There was no significant difference for Patent Ductus Arteriosus between two groups (*p* =1). Pneumothorax was seen in two patients of control group, but not detected in any neonates of salbutamol group (*p* = 0.149). The duration of hospitalization was shorter in interventional group than control group,18.63 ± 8.62 and 28.31 ± 18.17 days, respectively (*p* = 0.047). Respiratory failure was the cause of death in all of the expired infants. The mortality rate was greater in control group [8(33.3 %) VS 5(20.8 %)]; however, these differences between interventional and control groups was not significant (*p* = 0.330).

**Table 2:** Comparison of INSURE failure (need for Mechanical Ventilation), the duration of NCPAP, the Mechanical Ventilation (M.V.) and oxygen requirement between control and interventional groups

Variable	Surfactant with N.S. (*n* = 24)	Surfactant with Salb. (*n* = 24)	*p*
INSURE Failure *n*(%)	16(66.7 %)	10(41.7 %)	0.082
Duration of NCPAP (h)	69.50 ± 54.97	51.63 ± 48.77	0.316
Duration of M.V. (h)	38.12 ± 80.67	9.73 ± 18.57	0.145
Duration of oxygen therapy(h)	93.56 ± 134.77	60.21 ± 41.88	0.313

*N.S* normal saline, *Salb* salbutamol, *M.V* mechanical ventilation

## Discussion

The primary aim of this study was to assess whether the increase of absorption of lung fluid by administering Salbutamol in addition to Surfactant, could affect the clinical course of neonates with RDS. This study showed that newborns with RDS who were treated with intratracheal Salbutamol, accompanied by Surfactant had a lesser degree of NCPAP failure and the need for mechanical ventilation. Also the duration of NCPAP, mechanical ventilation and oxygen therapy were shorter in interventional group than the control group.

However this diffrencess were not significant, that may be related to low number of our population study groups. Since the demographic characteristics of newborns in interventional and control groups were not different these findings were probably related to the use of Salbutamol accompanied by Surfactant in the treatment of newborns with RDS. A previous study showed that β-agonists, besides decreasing lung fluid, played a great role in improving arterial blood gases [28]. Lung fluid absorption is primarily the effect of active Na + transport across the pulmonary epithelium derived from lung air spaces to the interstitium. Intra alveolar Na + extracted from airspaces to the interstitium through activation of Na+/K + −ATPase. Βeta-agonists can induce Na+/K + −ATPase pump [29].

Sakumaet al. have shown that β-agonists induce cAMP and subsequently increase lung fluid clearance [30]. There was one case with patent ductus arteriosus in control group, that was not significantly difference between two groups. The duration of hospitalization was less in interventional groups compared with the control group. A shorter duration of NCPAP or the mechanical ventilation and early feeding of these neonates may be associated with earlier discharge of neonates in interventional groups. In our study, however, the mortality rate was not significantly different between interventional and control groups, but the mortality rate in control group was greater than the salbutamol group. Because of the underdevelopment of the intensive care for extremely low birth weight (ELBW) in our institute, we did not enroll newborns with ELBW in our study; thus, it is a great limitation of our study.

## Conclusion

According to the result of this study, intratracheal administration of β-adrenergic (Salbutamol) accompanied by Surfactant can decrease the duration of NCPAP, NCPAP failure rate and reduce the need for mechanical ventilation in newborns with RDS.
